# Hepatocellular carcinoma incidentally detected at second hepatectomy for repeated colorectal liver metastasis in a patient with hepatitis C virus-related cirrhosis: a case report

**DOI:** 10.1186/s13256-016-0927-2

**Published:** 2016-05-27

**Authors:** Atsuo Kobayashi, Daisuke Morioka, Chizuru Matsumoto, Yasuhiko Miura, Masaru Miura

**Affiliations:** Department of Surgery, Yokohama Ekisaikai Hospital, 1-2 Yamada-cho, Naka-ku Yokohama, 231-0036 Japan

**Keywords:** Case report, Viral hepatitis, Cirrhosis, Colorectal cancer, Liver metastasis, Hepatocellular carcinoma

## Abstract

**Background:**

It has been reported that liver metastasis rarely occurs in a cirrhotic/hepatitic liver. Thus, coexistence of liver metastasis and hepatocellular carcinoma has been scarcely reported. To the best of our knowledge, there are no cases with hepatocellular carcinoma, which developed during an observational period after hepatectomy for colorectal liver metastasis, in the worldwide English literature. Here we present a case of hepatocellular carcinoma which occurred during a period between the first and second hepatectomy for repeated colorectal liver metastasis.

**Case presentation:**

A 65-year-old Japanese woman underwent rectal resection for advanced rectal cancer. Hepatitis C cirrhosis was diagnosed at that time and antiviral therapy was offered but rejected because of socioeconomic reasons. At the age of 68, she developed two colorectal liver metastases originating from the rectal cancer, which were treated by local ablation and partial hepatectomy. At the age of 71, solitary recurrent colorectal liver metastasis was observed adjacent to the previously ablated lesion in segment 4, and thus segmentectomy 4 was performed. During surgery, a small tumor in segment 8 was incidentally identified. Taking into account her history, the tumor was considered to be recurrent colorectal liver metastasis and it was extirpated by partial hepatectomy. However, the segment 4 tumor was diagnosed as recurrent colorectal liver metastasis on the basis of histological findings and the segment 8 tumor was diagnosed as hepatocellular carcinoma. Although she had a cut surface abscess postoperatively, she was discharged from hospital 21 days after the surgery and is currently doing well 18 months after the second hepatectomy. She is currently receiving interferon and ribavirin therapy to eliminate hepatitis C virus.

**Conclusions:**

If antiviral therapy was performed earlier for the present case and viral elimination was achieved, hepatocellular carcinoma might not have developed. This case reemphasizes the importance of antiviral therapy for preventing carcinogenesis of hepatocellular carcinoma in patients with viral hepatitis even if they have other cancers.

## Background

It has been reported that liver metastasis originating from extrahepatic malignancy rarely occurs in hepatitic or cirrhotic liver [1–6]. The liver is known to be the most common organ to which colorectal cancer (CRC) metastasizes and liver metastasis originating from CRC has been reportedly observed in 15 to 30 % of patients with CRC during their life-long clinical course [7, 8]. Although liver metastasis is less likely to occur in a cirrhotic/hepatitic liver than in a normal liver, it has been reported that 8 to 10 % of patients with CRC with viral hepatitis developed liver metastasis [2, 3]. The number of patients with CRC has been increasing [7] and patients with hepatitis or cirrhosis are often encountered in daily clinical practice, although the number of patients infected with the hepatitis virus has been decreasing [9]. Thus, the coexistence of colorectal liver metastasis (CRLM) and hepatocellular carcinoma (HCC), which usually occurs in an injured liver, is considered common but is hardly ever seen in daily clinical practice. The impression that the coexistence of CRLM and HCC is rare is supported by the fact that such cases have been seldom reported with only two cases reported in PubMed indexed literature [10, 11]. Furthermore, to the best of our knowledge, no cases with HCC, which developed during the observational period after hepatectomy for CRLM, have been found in the worldwide English literature. Here we present a case of HCC which developed during a period between the first and second hepatectomy for repeated CRLM.

## Case presentation

**Fig. 1:**
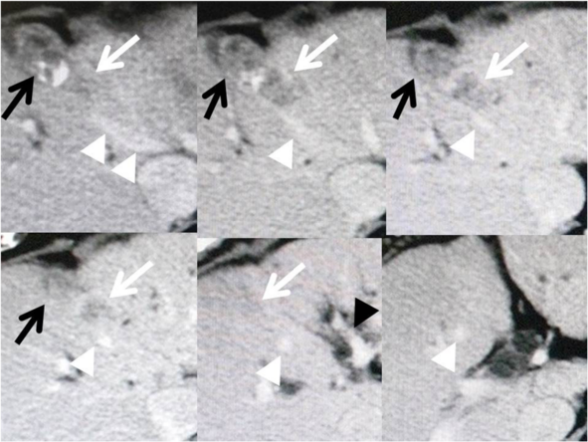
Computed tomography findings of the segment 4 tumor developing adjacent to the lesion previously treated with ablation. An irregularly shaped tumor (*white arrows*) was observed adjacent to the lesion previously treated with microwave tissue coagulation ablation (*black arrows*). An irregularly shaped tumor was broadly in contact with the middle hepatic vain (*white arrowheads*) and obviously separate from the umbilical portion of the portal vein (*black arrowhead*)

A 65-year-old Japanese woman, who was known to be infected with hepatitis C virus (HCV), underwent rectal resection for advanced rectal cancer, which represented serosal invasion and lymph node metastasis. In spite of HCV infection, the results of her liver function test were normal and she did not hope to receive antiviral therapy because of socioeconomic reasons. At the age of 68, she developed two CRLMs located in segments 4 and 6. She received neoadjuvant chemotherapy, including oxaliplatin, leucovorin, and fluorouracil, and subsequently underwent surgery composed of microwave tissue coagulator (MCT) ablation for segment 4 tumor and segmentectomy 6. The segment 4 tumor was considerably small with a diameter of 1 cm and it was exposed to the liver surface; thus, we performed MCT ablation. A histological examination of the resected S6 tumor revealed adenocarcinoma and was confirmed to be CRLM but not intrahepatic cholangiocarcinoma with negative cytokeratin 7 and positive cytokeratin 20 in immunohistochemistry. Although she was diagnosed histologically as having a cirrhotic liver, by a microscopic examination for background non-tumoral liver, antiviral treatment was again not conducted due to socioeconomic reasons at that time. At the age of 71, abdominal computed tomography (CT) showed a tumor that was adjacent to the previously ablated lesion in segment 4 of her liver (Fig. 1). The tumor was irregularly shaped and in contact with her middle hepatic vein (MHV). Based on a three-phase CT study, the tumor showed neither early enhancement nor washout and was less enhanced compared to non-tumoral background liver through all phases. Furthermore, no other tumor was detected in any phases of the three-phase study. Based on these findings and her previous history of rectal cancer liver metastases, we considered the segment 4 tumor to be a recurrent liver metastasis originating from rectal cancer. Because her liver function was well preserved and corresponded to Child-Pugh A (5 points), surgery was undertaken. During the surgery, a small tumor was identified in segment 8, which could not be diagnosed preoperatively. Taking her history into account, we considered that the segment 8 tumor was also a recurrent CRLM. The segment 4 tumor was broadly in contact with her MHV and less distinguishable from the lesion previously treated by MCT. Thus we performed segmentectomy 4 including MHV for the segment 4 tumor. With regard to the segment 8 tumor, it was exposed to the liver surface and well recognizable. Hence, we considered applying MCT ablation to the segment 8 tumor. However, the segment 4 tumor, which was resected at that time, might have been a local recurrence of the segment 4 tumor previously treated by MCT ablation. In other words, MCT ablation might not have been effective enough or might have induced tumor implantation into her liver even if the tumor was small and exposed to the liver surface. Thus we performed partial hepatectomy for segment 8 tumor; methods of hepatectomy were described according to the Brisbane 2000 system of nomenclature of liver anatomy and resections [12].

**Fig. 2:**
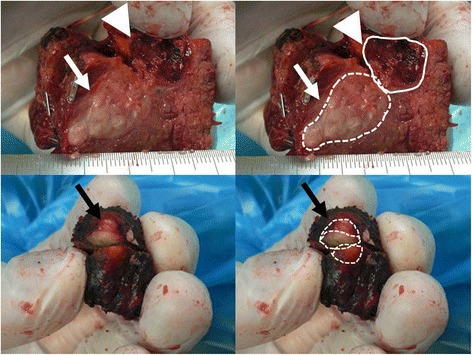
Macroscopic findings of the segment 4 and 8 tumors. Upper left, segment 4 tumor. An irregular shaped tumor (*white arrows*) was seen adjacent to the lesion previously treated with ablation (*white arrowheads*). Upper right, an irregular shaped tumor is outlined with *white dotted line* and previously ablated lesion with *white line*. Lower left, segment 8 tumor. A small white spherical tumor (*black arrow*) was seen on the surface of the specimen. Lower right, segment 8 tumor is outlined with *white dotted line*

**Fig. 3:**
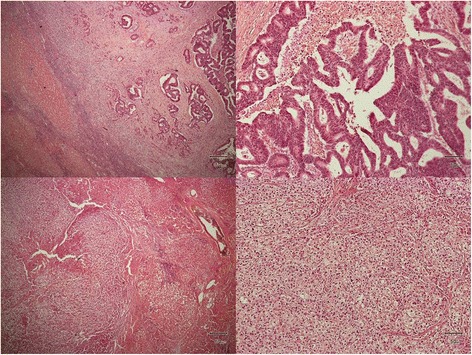
Histopathological findings of the tumors. *Upper part*, histological findings of the segment 4 tumor. *Upper left*, a stroma-rich tumor was seen in the cirrhotic liver (hematoxylin and eosin stain; original magnification, ×40). *Upper right*, moderate disorder of the tubuloglandular structures was observed and cellular polarity was no longer maintained, suggesting that segment 4 tumor was composed of moderately differentiated adenocarcinoma (hematoxylin and eosin stain; original magnification, ×200). *Lower part*, histological findings of the segment 8 tumor. *Lower left*, a tumor with little stroma was seen in the cirrhotic liver (*left half* of the picture; hematoxylin and eosin stain; original magnification, ×200). *Lower right*, structural atypia was mild and cellular polarity was relatively maintained although sizes of the nuclei were varied in this tumor. The segment 8 tumor was diagnosed as well-differentiated hepatocellular carcinoma based on these findings (hematoxylin and eosin stain; original magnification, ×200)

Histological findings showed a moderately differentiated adenocarcinoma in the segment 4 tumor, confirming that the segment 4 tumor was a recurrent rectal cancer liver metastasis as diagnosed preoperatively (Fig. 2). The diagnosis of recurrent CRLM was corroborated by the immunohistochemistry that showed negative for cytokeratin 7 and positive for cytokeratin 20, indicating that the segment 4 tumor was CRLM but not intrahepatic cholangiocarcinoma that often develops in cirrhotic liver. However, histology of the segment 8 tumor showed findings of well-differentiated HCC (Fig. 2). According to the Metavir system [11], we determined the inflammation and fibrosis status of the non-tumoral background liver to be A1F4 (Fig. 3). As to the HCC, it was classified as early stage (A) of the Barcelona Clinic Liver Cancer staging system because the tumor was solitary and 1 cm in size, the liver function of the patient corresponded to Child-Pugh A, and performance status corresponded to 0 [13].

**Fig. 4:**
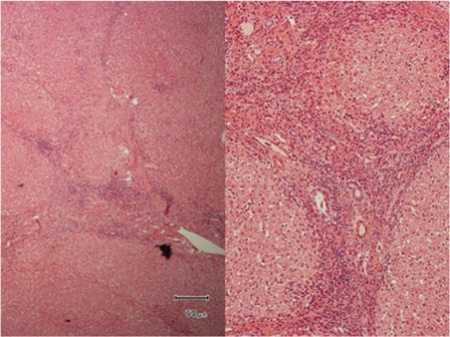
Histological findings of the non-tumoral background liver. *Left*, remarkable disorder of the lobular structure caused by the bridging fibrosis was observed (hematoxylin and eosin stain; original magnification, ×40). *Right*, little interface hepatitis was seen and necro-inflammatory reaction was observed only slightly. Based on these findings, we judged the condition of the non-tumoral background liver to be A1F4 according to the Metavir system

**Fig. 5:**
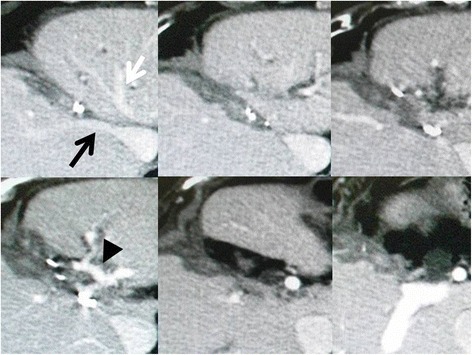
Computed tomography findings after the segment 4 segmentectomy. Although the right (*black arrow*) and left (*white arrow*) hepatic veins were recognizable, the middle hepatic vein was not observed. Furthermore, liver parenchyma right and adjacent to the umbilical portion of the portal vein (*black arrowhead*) was not identified. These findings suggested that segmentectomy 4 including the middle hepatic vein was properly achieved

Although she had a cut surface abscess postoperatively, she was discharged from hospital 21 days after the surgery and is currently doing well 18 months after the second hepatectomy (Fig. 4). She is currently receiving interferon and ribavirin therapy to eliminate HCV (Fig. 5).

## Discussion

A recent report showed that liver metastasis risk was underestimated and it was even higher in patients with CRC with cirrhosis of the liver [14]. In the report, however, neither diagnostic modalities for liver metastasis nor occurrence of HCC were specified. Namely there is a possibility of misdiagnosing HCC as CRLM. Thus the idea has been gaining relatively firm evidence; therefore, it is being accepted that CRLM is less likely to occur in patients with hepatitis or cirrhosis than in patients with a normal liver [4–6]. Although the reason for the rare occurrence of liver metastasis in diseased liver has not been explained clearly, it is considered that persistent inflammation in hepatitic/cirrhotic liver is unfavorable for the implantation of cancer cells, originating from extrahepatic organs, in hepatic tissue [1–8]. If this hypothesis is true, it can be stated that the less severe the inflammation is, the more likely that liver metastasis will occur. In this scenario, the present case showed normal liver function test and histology of non-tumoral background liver represented A1 and F4 of Metavir system [11], suggesting that her inflammation is mild and less unfavorable for cancer cell implantation. However, mild but persistent inflammation and liver fibrosis certainly existed and contributed to carcinogenesis of HCC. Furthermore, some authors reported that only if hepatic function is preserved, would patients with an injured liver who underwent curative resection for CRC show better prognosis than those with an uninjured liver [3, 5]. In addition, with the progression of hepatectomy procedure and chemotherapy, the prognosis of patients with CRC has been improving even in patients with CRLM [15]. Therefore, patients with hepatitis or cirrhosis with CRC could gain long-term survival after curative treatment if hepatic function is well preserved. In this patient population, hence, management of background hepatic disease is important as well as surveillance for CRC recurrence to obtain further long-term survival. In the present case, antiviral therapy was not conducted after the rectal resection although she was confirmed to be infected with HCV. Furthermore, we recognized that the background non-tumoral liver of the patient was cirrhotic at the first hepatectomy; nevertheless, antiviral therapy was not performed although she did not hope to receive it. Consequently, she developed HCC. If viral elimination was achieved after the first hepatectomy, HCC may not have developed. The clinical course of the present case corroborates that it is very important to eliminate virus for patients with cirrhosis with HCV in terms of preventing development of HCC even if hepatic function is well preserved, and reemphasizes that management of liver disease, including surveillance of HCC occurrence, is important as well as surveillance of CRC recurrence in patients with hepatitis or cirrhosis with CRC.

When a liver tumor is diagnosed in patients with cirrhosis or hepatitis who have a history of CRC, specification of the diagnosis for liver tumor is very important because treatment strategy varies according to the diagnosis [7–9, 13, 15]. Recently, contrast-enhanced ultrasound and/or contrast-enhanced magnetic resonance imaging have been reported to be superior to contrast-enhanced CT in terms of diagnostic ability for detection and discrimination of liver tumors [16]. In the present case, therefore, these imaging studies should have been performed in addition to CT for distinguishing between CRLM and HCC more clearly and detecting other lesions that could not be detected by CT, although preoperative diagnosis of CRLM for CT-proven liver tumors was fortunately correct.

## Conclusions

We present a case of HCC developing during a period between the first and second hepatectomy for repeated CRLM. With the experience of this case, we must realize that management of liver disease, including surveillance of HCC occurrence and virus elimination, is markedly important as well as surveillance of CRC recurrence in patients with hepatitis or cirrhosis with CRC.

## Abbreviations

CRC: colorectal cancer
CRLM: colorectal liver metastasis
CT: computed tomography
HCC: hepatocellular carcinoma
HCV: hepatitis C virus
MCT: microwave tissue coagulator
MHV: middle hepatic vein
